# Mr. W. Sharp, on the Cow-Pox

**Published:** 1801-02

**Authors:** W. Fermor, Wm. Sharp

**Affiliations:** Tusmore; Wicken Park, Bucks


### *To the Editors of the Medical and Physical Journal.*

Gentlemen,

As I am very certain the contents of the following letter from Mr. W. Sharp, who is now retired from business, on the subject of the Cow-pox and its origin, is interesting, I wish much to have it inserted in the Medical and Physical Journal. I am, &c.

*Tusmore, Jan.* 16, 1801. W. FERMOR.

---

" Dear Sir,

" When I had the pleasure of meeting you the other morning, you asked me, if the Cow-pox prevailed amongst the cattle tle in the North of England? I could not then anfwer your queftion; but I enquired, and was informed by a letter this morning from Durham, that the difeafe is not known there among the cattle; and alfo, that they had heard of it in four counties only, namely, Gloucefter, Oxford, Bucks, and Northamptonfhire.*

"From circumftances which have fallen in my way, I believe that the diforder arifes from infection brought to the cows by hands employed about the difeafed heels of horfes; and in confequence, that it could not be known there, as the cows are univerfally milked by women, who have nothing to do in the ftable.

"Wifhing all poffible fuccefs to the Inoculation of the Cow-pox, which you have fo meritorious a fhare in promoting, I am,

"Dear Sir,

"Your's, &c.

"WM. SHARP."

*Wicken Park, Bucks,*

*Dec.* 18, 1800

---

### *A Case of strangulated Crural Hernia, where the Operation succeeded after the Obstruction had continued Six Days.*

By G. BORRETT, Surgeon, Great Yarmouth, and Member of the Royal College of Surgeons, London.

---

### *To the Editors of the Medical and Phyfical Journal.*

* It has been obferved alfo in Somerfetfhire, Devonfhire, Wiltfhire, Berks, and fome others.

